# *Nelumbo nucifera* Gaertn Stems (Hegeng) Improved Depression Behavior in CUMS Mice by Regulating NCAM and GAP-43 Expression

**DOI:** 10.1155/2020/3056954

**Published:** 2020-03-30

**Authors:** Ya-Nan Zhao, Ya-Fei Cao, Yan-Hong Zhang, Ye Lu, Xin Ping, Shao-Kun Qin, Shu-Ning Liu, Li Chu, Guo-Qiang Sun, Lin Pei

**Affiliations:** ^1^School of Chinese Medicine, North China University of Science and Technology, Tangshan, Hebei 063210, China; ^2^Hebei University of Chinese Medicine, Shijiazhuang, Hebei 050051, China; ^3^Hebei Key Laboratory of Turbidity, Hebei Academy of Chinese Medicine Sciences, Shijiazhuang, Hebei 050011, China; ^4^School of Chinese Medicine, Hebei University, Baoding, Hebei 071051, China

## Abstract

**Background:**

*Nelumbo nucifera* Gaertn stem (Hegeng [HG]) is a traditional Chinese medicine that is used to treat mental symptoms in East Asia. However, scientific evidence is generally lacking to support this traditional claim. *Aim of the Study*. This study's aim is to investigate the antidepression effect of HG and to further explore the possible molecular mechanisms that are involved in its actions. *Materials and Methods*. HG aqueous extract was administered intragastrically for 21 days after the chronic unpredictable mild stress (CUMS) procedure, and its effect on memory, learning, and emotion was assessed using animal behavioral tests. HG aqueous extract was characterized using HPLC. Immunofluorescence was used to measure the neural cell-adhesion molecule (NCAM) and growth-associated protein-43 (GAP-43) expression.

**Results:**

Depression-like behaviors increased in the CUMS group compared with the control (CON) group, while they were reduced in the high-dose HG (H-HG) and fluoxetine (FLU) groups (*p* < 0.05). Additionally, NCAM and GAP-43 expression was reduced in the CUMS group compared with the CON group, but it increased in the H-HG and FLU groups (*p* < 0.05).

**Conclusions:**

These findings show the potential antidepressant effects of HG through mechanisms involving regulation of NCAM and GAP-43. This provides a new theoretical basis for its potential application as an antidepressant-like agent.

## 1. Introduction

Depression is one of the most common severe psychiatric conditions [1]. Depression symptoms include depressed mood, reduced or increased craving for food, irritability, and loss of energy [2]. The World Health Organization estimated that depressive disorders will be an important contributor to the global burden of diseases by 2030 [3].

Learning and memory are advanced cognitive functions in the brain [4], and these are popular areas of research in psychology. The centralization of learning and memory ability, synaptic plasticity and various biologically active substances, and the synthesis of new proteins that play a crucial role in the process of memory and learning ability have been extensively studied [5]. In the central nervous system (CNS), the hippocampus is the key brain region that regulates memory and learning ability, and it is also the main target organ of the stress response; thus, the hippocampus is susceptible to stress [6]. Different hippocampal regions in mice have different involvement in the process of spatial learning and memory, and impaired CA1, CA3, and DG will cause difficulties in spatial learning (such as in the Morris water maze [MWM] task) [7].

Recently, the role of neural cell-adhesion molecule (NCAM) and growth-associated protein-43 (GAP-43) in learning and memory has been investigated [8]. NCAM is a membrane glycoprotein that mediates cell adhesion and recognition, and it affects the formation of neural pathways, signal transmission processes, nerve development, injury repair, and other neurophysiological processes, especially in terms of synaptic plasticity, formation, and consolidation of memory and learning ability [9]. GAP-43 is a neural tissue-specific presynaptic membrane phosphoprotein that is bound by calmodulin, which is closely related to axonal growth, synaptic formation and remodeling, neurotransmitter release, and other processes, and it is considered to be a marker of neuron development and plasticity [10]. NCAM and GAP-43 play a synergistic role when axonal growth is stimulated. Previous research concentrated more on their learning memory and brain damage repair, and more recent research shows that NCAM and GAP-43 are involved in the stress response process [11]. These results provide novel observations, which indicate that NCAM and GAP-43 may have a significant effect on the mechanisms of antidepressants.

However, reported antidepressant therapies have undesirable related adverse side effects [12]. Therefore, the search for new antidepressants with no or fewer side effects continues and some herbal medicines could be successful alternatives for treating patients with depression [13,14].

*Nelumbo nucifera* Gaertn (Hegeng [HG]) stem is a traditional Chinese medicine (TCM). According to ancient Chinese medical books, such as “A Guide to Clinical Practice with Medical Record, Lin Zheng Zhi Nan Yi An,” the function of HG is to soothe the liver and regulate qi (the life force energy the Chinese call “qi” [15]) by alleviating mental depression. Clinically, HG is prescribed in combination with other Chinese herbal medicines to treat mental depression resulting from liver-qi stagnancy, based on the TCM theory of “Gan-zhu-shu-xie” (liver controlling conveyance and dispersion). Moreover, our experimental studies also have demonstrated that Chinese medicine compound preparations containing HG can exert antidepressant effects via activating the Brain-Derived Neurotrophic Factor (BDNF)-Rac1-cofilin signal pathway [16]. However, little is known about HG's antidepressive effects and mechanisms. We hypothesized that HG ameliorates chronic unpredictable mild stress (CUMS) depressive behaviors. In the current experiments, we explored the antidepressant-like effects of HG on learning and memory behaviors as well as possible mechanisms using the CUMS mouse model.

## 2. Materials and Methods

### 2.1. Plant Material and Extraction

HG stems were purchased from the Affiliated Hospital of Hebei Academy of Traditional Chinese Medicine and were processed according to the Chinese Pharmacopoeia [17] before extraction. The identity of the HG stems was confirmed by Xiaogang Wang at the Affiliated Hospital of Hebei Academy of Traditional Chinese Medicine (Shijiazhuang, Hebei, China). Voucher specimens (N201010) were deposited at the Affiliated Hospital of Hebei Academy of Traditional Chinese Medicine. The stems were air-dried and were reduced to the proper size. The plants were boiled after soaking in 800 mL of pure water for 35 minutes. Then the extract was filtered using cotton gauze and followed by Whatman filter paper No. 1. Finally, the extract was freeze-dried for 1 week and was resuspended in 300 mL of distilled water until use.

### 2.2. Drugs and Reagents

HG was obtained from the Affiliated Hospital of Hebei Academy of Traditional Chinese Medicine. Fluoxetine (Prozac®, Suzhou, China) was obtained from Patheon France. The reference substance standard Nuciferine (purity > 98%) was purchased from Beijing Zhongke Zhijian Biotechnology Co., Ltd. (Beijing, China). Mouse anti-NACM1 polyclonal antibody (RNL-1, Abcam, Cambridge, UK) and rabbit anti-GAP43 polyclonal antibody (EP890Y, Abcam) were purchased from Hebei Bio-High Technology Co., Ltd. (Shijiazhuang, China).

### 2.3. Animals

Forty Institute of Cancer Research (ICR) male mice (age, 6-7 weeks; weight, 25–30 g) were obtained from the Animal Experiment Center of Hebei Medical University, Hebei, China (license number SCXK 2018-004, certificate number 1808129). All mice were housed individually in cages (39 × 23 × 16 cm) for 3 weeks and with a 12 h light/dark cycle (lights on 7:00 a.m–7:00 p.m) with unrestricted access to food (Animal Experiment Center of Hebei Medical University, Hebei, China; license number SCXK 2018-003) and tap water. All experiments were performed at a controlled temperature (24 ± 2°C) and humidity (60 ± 1%). Experiments were performed in compliance with the National Institutes of Health Guide for the Care and Use of Laboratory Animals. All animal experiments were approved by the Institutional Animal Care and Use Committee at Hebei Academy of Traditional Chinese Medicine and complied with the Animal Management Rules of the Chinese Ministry of Health and existing current animal welfare guidelines.

### 2.4. Groups and Drug Administration

The mice were randomly divided into five groups (*n* = 8 animals/group, on average) as follows: control (CON), CUMS, fluoxetine (FLU) (10 mg/kg), high-dose HG (H-HG, 400 mg/kg), and low-dose HG (L-HG, 200 mg/kg) groups. All treatments were administered to the mice via gastric gavages except for mice in the CON group. The CON group was treated normally, while the FLU group was given fluoxetine hydrochloride and the H-HG and L-HG groups were given the associated HG dose for 3 weeks. The CUMS group received saline vehicle via gavage. The HG dosages selected in this study were based on the clinical therapeutic doses of Chinese medicine compound preparations containing HG for the treatment of depression [18].

### 2.5. CUMS Protocol

**Figure 1:**
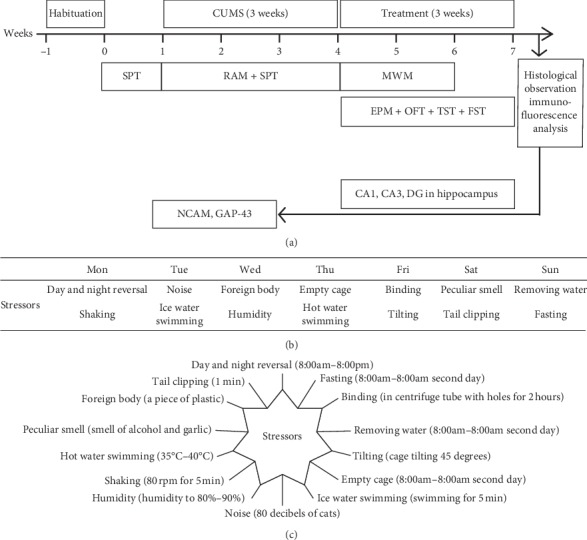
Flow chart depicting the procedural order used in this experiment (a). Chronic unpredictable mild stress protocol (b). Description of stressors (c).

The CUMS protocol is shown in Figure 1. The major depressive-like behaviors of this mouse model were induced by CUMS. The CUMS protocol was based on other studies [19]. After habituation, mice were exposed to unpredictable mild stressors for 21 d in this protocol; we used 14 diverse stressors and two stressors were applied per day. The stressors included day and night reversal, fasting, binding, removing water, tilting, empty cage, ice water swimming, noise, shaking, humidity, thermal stimulation, peculiar smell, clipping, and foreign body. Mice in the control group were handled without any stress.

### 2.6. HPLC Analysis

A Waters-1525 high performance liquid chromatography (HPLC) system was used to detect the HG aqueous extract. HPLC conditions were as follows: Kromasil C18 column (250 × 4.6 mm, 5.0 *μ*m, Nobel®, Gothenburg, Sweden) was used with an acetonitrile : water : triethylamine : glacial acetic acid (27 : 70.6 : 1.6 : 0.78) mixture as a mobile phase; the flow rate was 1.0 mL/min; and the column temperature was 25°C. The volume of HG aqueous extract was 2 *μ*L, and nuciferine was detected at 270 nm.

### 2.7. Immunofluorescent Staining of NCAM and GAP-43

After the behavioral tests, all the mice were fully anesthetized intraperitoneally with a 2% sodium pentobarbital (Huaye Huanyu Chemical Co. Ltd., Beijing, China). Mice were perfused through the heart with a 0.9% sodium chloride solution followed by 4% paraformaldehyde, and then the brains were fixed in 4% paraformaldehyde for 48 h and dehydrated in 30% sucrose at 4°C overnight. The next day, these brains were embedded in paraffin and 5 mm thick sections were cut for immunofluorescence. The tissue sections were incubated in 1% bovine serum albumin for 1 h and then with the following primary antibodies at 4°C overnight: rabbit GAP-43 (diluted 1 : 50) and mouse anti-NCAM (diluted 1 : 50). The sections were then rinsed five times each for 5 min with phosphate buffered saline (PBS) and incubated for 2 h with the following secondary antibodies: anti-mouse IgG-TRITC and goat anti-rabbit IgG-Alexa Fluor-488 (diluted 1 : 50). Finally, the samples were washed five times each in PBS. Tissue sections were placed under a coverslip for fluorescence detection.

## 3. Animal Behaviors

All mouse behaviors were detected and documented using the animal behavioral activity record analysis system (SLY-ETS-WIN7, Beijing Sunny Instruments Co. Ltd., Beijing, China).

### 3.1. Sucrose Preference Test

The sucrose preference test (SPT) was performed to measure depression-like behavior. The test was performed according to previously reported papers, with a slight modification [20]. Each mouse was adapted to two bottles of 1.0% sucrose solution for 24 h and then exposed to two bottles for an additional 24 h, where one bottle contained tap water and the other contained 1.0% sucrose solution in tap water. After 24 h, the sucrose solution in one of the bottles was substituted with pure water. Then, mice were given the following two bottles for 24 h: tap water and 1% sucrose solution. The sucrose preference rate was analyzed by weighing the bottle of tap water and 1% sucrose solution.

### 3.2. Radial Arm Maze Test

The learning and memory ability task was performed in the radial arm maze (RAM). The RAM test was performed according to previously published methods, with a slight modification [21]. Briefly, the central platform had a diameter of 16 cm with 30 cm length × 8.5 cm width of arms projecting radially outwards. The apparatus was placed in a dimly lit room and elevated to 60 cm above the floor. Sweet food pellets were placed at the end of four arms (1, 2, 6, 7). To ensure adequate motivation in this maze test, the mice were kept on food restriction (85% of their regular diet was withheld). During the habituation phase, the mice were allowed to become familiar with the RAM equipment by two 15 min trials each day. On the first day, the mice were free to probe the apparatus and food was spread all over the arms. On days 2–5, only the chambers at the end of arms had food so that the mice could probe the chambers to reach the food. The testing time was 5 min and the same arms (1, 2, 6, 7) remained baited to habituate the mice for the trials. Each trial started by placing the mice onto the central platform, after which the sliding doors were lifted slowly, and the mouse was allowed to enter any arm to explore the maze. Each session lasted for 5 min. We performed an acquisition phase consisting of one trial per session for 7 consecutive days and a retention test at 14, 21, and 28 days after the last session. Baited arms were counted as “working memory error” [22] and the time to consume the four pellets was recorded as the “residence time of correct feeding arms.”

### 3.3. Morris Water Maze Test

Mice were trained to study the position of a hidden platform using a standardized Morris water maze (MWM) test process to monitor and evaluate their spatial learning and memory [23]. The test included place navigation and a spatial probe test. Mice were first placed into the water maze (height, 60 cm; diameter, 120 cm), which was divided into four quadrants and maintained at 20 ± 2°C by an automatic heater. The water was made opaque using black ink and a platform was placed into the water. During the last 6 days, each mouse received one session of 1, 2, 3, and 4 training trials per day. For four trials per day, the mice were placed at diverse starting positions in a specific order (1, 2, 3, and 4 or 4, 3, 2, and 1); the platform was placed in quadrant 3. The mouse was released in the water from one of the four starting positions. When the mouse reached the platform, it was given 120 s to explore the latent platform, and its escape latency was considered to be 120 s. After the last test, the platform was removed from the water and the mice performed an uncontrolled probe for 1 min to evaluate their learning and memory ability to search the platform [24].

### 3.4. Elevated Plus Maze Test

The elevated plus maze (EPM) was performed according to previous studies [3,25]. The device consists of two closed arms (30 × 5 × 15 cm) and two open arms (30 × 5 × 15 cm). The mouse was introduced to the center (6 × 6 cm) of the plus maze facing the same arm for each test and was allowed to explore for 5 min. Entries into open arms, distance in open arms, and time in open arms were quantified as indices of depressive behavior.

### 3.5. Open-Field Test

The open-field test (OFT) has been reported to examine the state of independent activity [26]. Reduction of locomotor activity is a characteristic symptom of major depression [27]. The test room was dimly illuminated (40 W) and the OFT test was conducted in a black device (60 × 60 cm) and allowed to roam freely for a testing duration of 5 min. Before the test, mice were permitted to explore the areas freely for 1 h. The time at the center, the number of entries into the center, and the distance to the center were recorded using a high-definition camera.

### 3.6. Tail Suspension Test

The tail suspension test (TST) is routinely used to determine the level of desperation and helplessness in mouse [28]. Each mouse was suspended separately by having its tail fixed onto a rod and taping its tail with adhesive Scotch tape. The test lasted for 6 min and the duration of immobility was measured during the last 4 min. In the TST, immobility time was tested as an index of depressive behavior.

### 3.7. Forced-Swimming Test

The forced-swimming test (FST) has been reported to measure the extent of behavioral despair in the CUMS model. The text was conducted according to previously established protocols [29]. The mice swam in a glass beaker filled with water at 24°C for 15 min before testing [30]. During the test, mice were placed separately into the glass beaker for 6 min. The immobility time and latency to immobility time were quantified as indices of depressive-like behavior. The definition of immobility was when mice did not make any movements or only made movements to maintain balance.

## 4. Statistical Analysis

The data obtained were expressed as the mean ± standard error of the mean (SEM) and were analyzed by one-way analysis of variance (ANOVA), followed by a Student's *t*-test using Origin Pro 9.1 software. Groups were compared using Fisher's LSD test. *p* < 0.05 was considered as statistically significant.

## 5. Results

### 5.1. Content Analysis of HG by HPLC

**Figure 2:**
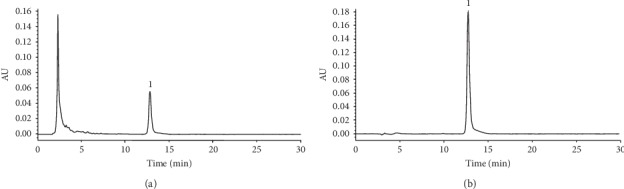
HPLC profiles of HG aqueous extract (a) and standards (b). Peak 1, nuciferine.

Nuciferine was a main component of HG aqueous extract as analyzed using HPLC (Figure 2). Results of the analysis showed that 0.66 mg/mL of nuciferine was administered in the HG aqueous extract.

### 5.2. Effects of HG in SPT Test

**Figure 3:**
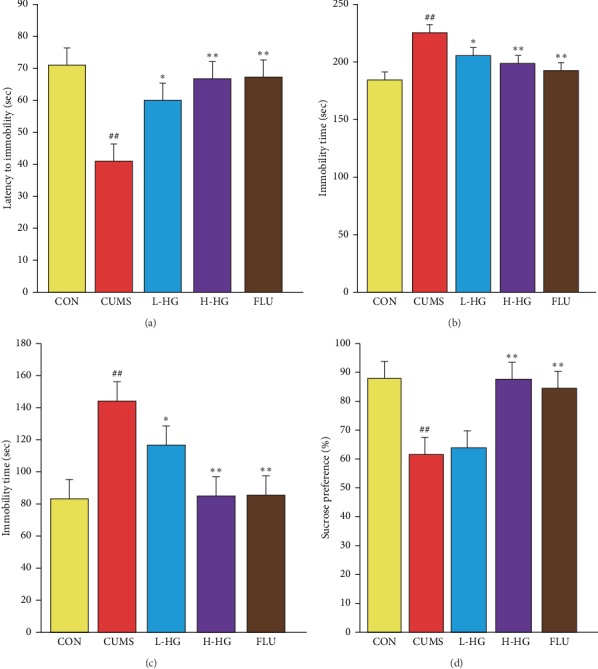
Effects of HG on depressive behaviors in the CUMS mouse model. Latency to immobility (a) and immobility time in FST (b). Immobility time in TST (c), and sucrose preference in SPT (d). All values are expressed as the mean ± SEM (*n* = 8). ^*∗*^*p* < 0.05 and ^*∗∗*^*p* < 0.01 compared to the CUMS group; ^#^*p* < 0.05 and ^##^*p* < 0.01 relative to the CON group.

The SPT results are shown in Figure 3(d). Compared to the CON group, the CUMS group showed significantly less sucrose intake (*p* < 0.01). We next analyzed the three groups for their responses to drug treatment. Compared with the CUMS group, the sucrose intake was increased in mice in the H-HG and FLU group (*p* < 0.01).

### 5.3. Effects of HG in RAM Test

**Figure 4:**
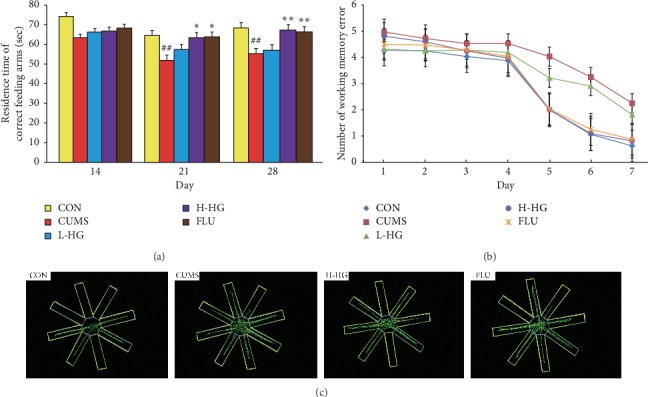
Effects of HG on mouse behavioral evaluated parameters in RAM. Residence time in the correct feeding arms (a), number of working memory errors (b), and graphic representation of the RAM movement (c). All values are expressed as the mean ± SEM (*n* = 8). ^*∗*^*p* < 0.05 and ^*∗∗*^*p* < 0.01 compared to the CUMS group; ^#^*p* < 0.05 and ^##^*p* < 0.01 relative to the CON group.

The RAM results are shown in Figure 4. RAM analysis showed the numbers of working memory errors and the residence time in the correct feeding arms as a reference. Compared to the CON group, working memory errors (at 7 days) increased (*p* < 0.01). The number of working memory errors in the CUMS group was increased significantly compared to the FLU (*p* < 0.01), H-HG (*p* < 0.01), and L-HG (*p* < 0.05) groups. Compared to the CON group, the residence time in the correct feeding arms (at 21 days) was shortened (*p* < 0.01) and the residence time in the correct feeding arms (at 28 days) was shortened (*p* < 0.01) in mice in the CUMS group. The residence time in the correct feeding arms (at 21 days) in the CUMS group was decreased significantly compared to the FLU and H-HG groups (*p* < 0.05) and the residence time in the correct feeding arms of the FLU and H-HG groups (*p* < 0.01) was significantly increased compared with the CUMS group (at 28 days).

### 5.4. Effects of HG in MWM Test

**Figure 5:**
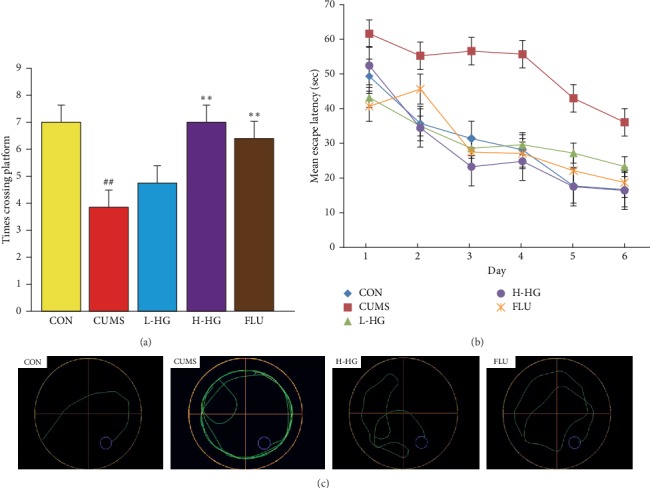
Effects of HG on mouse behavioral parameters that were evaluated in the MWM. The platform was centered in the southeast quadrant. Times crossing the platform (a) in spatial probe stages and the mean escape latency (b) in the hidden platform task. Graphic representation of the MWM movement (c). All values are expressed as the mean ± SEM (*n* = 8). ^*∗*^*p* < 0.05 and ^*∗∗*^*p* < 0.01 compared to the CUMS group; ^#^*p* < 0.05 and ^##^*p* < 0.01 compared to the CON group.

The MWM results are shown in Figure 5. Compared to the CON group, the platform area crossing time was reduced (*p* < 0.01), and the escape latency in the sixth test was longer (*p* < 0.01) in mice from the CUMS group. The platform area crossing time in the CUMS group was lower compared to the FLU and H-HG groups (*p* < 0.01). The mean escape latency in the sixth test in the CUMS group was longer than that of the FLU (*p* < 0.01), H-HG (*p* < 0.01), and L-HG (*p* < 0.05) groups.

### 5.5. Effect of HG in EPM Test

**Figure 6:**
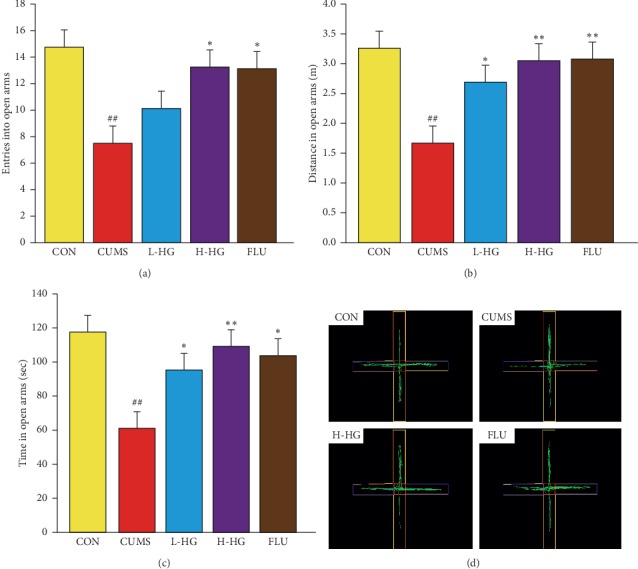
Effects of HG on depression-like behaviors in EPM. Entries into the open arms (a), distance in the open arms (b), time in the open arms (c), and graphic representation of the EPM movement (d). All values are expressed as the mean ± SEM (*n* = 8). ^*∗*^*p* < 0.05 and ^*∗∗*^*p* < 0.01 compared to the CUMS group; ^#^*p* < 0.05 and ^##^*p* < 0.01 compared to the CON group.

The EPM results are shown in Figure 6. Compared to the CON group, the number of entries into the open arms, the distance in the center, and the time spent in the center were decreased among mice in the CUMS group (*p* < 0.01). Compared to the CUMS group, entries into open arms showed an expected decrease in the FLU and H-HG groups (*p* < 0.05); the distance in the center and the time spent in the center showed an expected decrease in the FLU (*p* < 0.05) and H-HG groups (*p* < 0.01).

### 5.6. Effect of HG in the OFT Test

**Figure 7:**
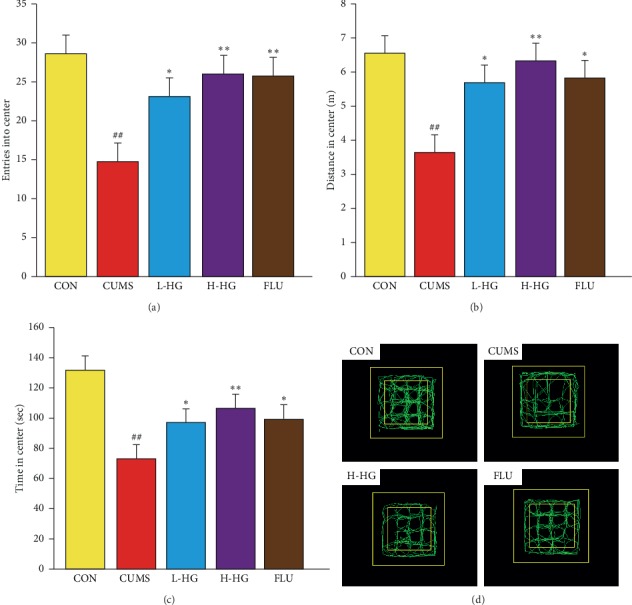
Effects of HG on depression-like behaviors in OFT. Entries into the center (a), distance in the center (b), time in the center (c), and graphic representation of the OFT movement (d). All values are expressed as the mean ± SEM (*n* = 8). ^*∗*^*p* < 0.05 and ^*∗∗*^*p* < 0.01 compared to the CUMS group; ^#^*p* < 0.05 and ^##^*p* < 0.01 compared to the CON group.

The OFT results are shown in Figure 7. In this experiment, compared to the CON group, the time spent in the center, the distance in the center, and entries into the center were reduced in mice from the CUMS group (*p* < 0.01). Compared against the CUMS group, entries into center, the time spent in center, and the distance in center showed an expected decrease in the FLU group, the H-HG, and L-HG groups (*p* < 0.05).

### 5.7. Effect of HG in the TST Test

The TST results are shown in Figure 3(c). In the TST, compared to the CON group, the immobility time was increased in the CUMS group (*p* < 0.01). The immobility time in the CUMS group was significantly increased compared to the FLU (*p* < 0.01), H-HG (*p* < 0.01), and L-HG groups (*p* < 0.05).

### 5.8. Effect of HG in the FST Test

The FST results are shown in Figures 3(a) and 3(b). In the FST, compared to the CON group, the latency to immobility and the immobility time were increased in the CUMS group (*p* < 0.01). The latency to immobility in the CUMS group was significantly reduced compared to the FLU, H-HG (*p* < 0.01), and L-HG groups (*p* < 0.05). The immobility time in the CUMS group showed a remarkable increase compared to the FLU (*p* < 0.01), H-HG (*p* < 0.01), and L-HG groups (*p* < 0.05).

### 5.9. Effects of HG on NCAM and GAP-43 Protein Expression in the Hippocampus Assessed by Immunofluorescence Analysis

**Figure 8:**
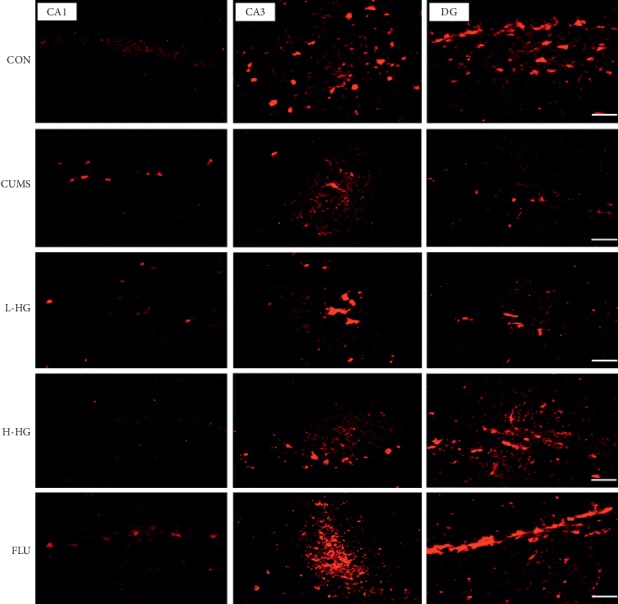
Representative immunohistofluorescent images of NCAM in the hippocampal CA1, CA3, and DG regions. Scale bar shows 200 *μ*m.

**Figure 9:**
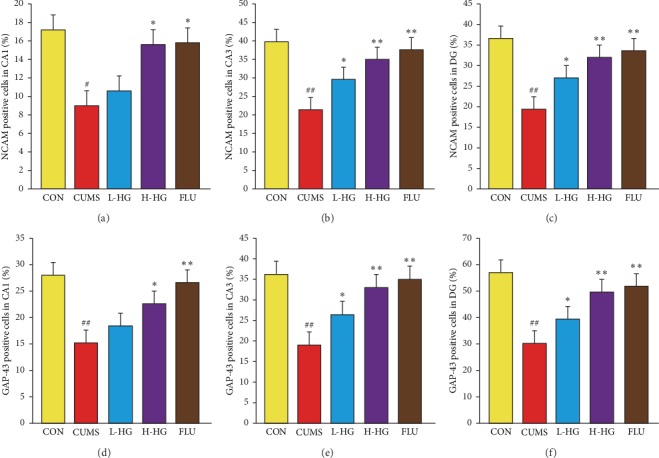
Results of immunohistofluorescent (IHF) analysis on NCAM and GAP-43 protein expression. The percent of NCAM-positive cells in the hippocampal CA1, CA3, and DG regions (a)–(c). The percent of GAP-43-positive cells in the hippocampal CA1, CA3, and DG regions (d)–(f). All values are expressed as the mean ± SEM (*n* = 5). ^*∗*^*p* < 0.05 and ^*∗∗*^*p* < 0.01 compared to the CUMS group; ^#^*p* < 0.05 and ^##^*p* < 0.01 compared to the CON group.

**Figure 10:**
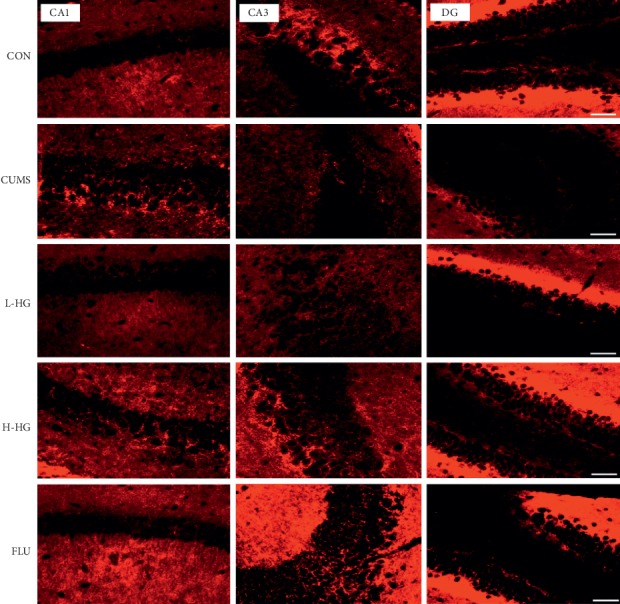
Representative immunohistofluorescent images of GAP-43 in the hippocampal CA1, CA3, and DG regions. Scale bar shows 200 *μ*m.

HG increased NCAM and GAP-43 protein expression in the hippocampus in the animal model of depression. As shown in Figures 8 and 9(a)–9(c), compared to the CON group, the proportion of NCAM-positive cells in the hippocampal CA1, CA3, and DG regions in the CUMS group was decreased (*p* < 0.05). The percent of NCAM-positive cells in the hippocampal CA1, CA3, and DG regions in the CUMS group was lower than that of the FLU group and the H-HG group (*p* < 0.05). As shown in Figures 9(d)–9(f) and 10, compared to the CON group, the proportion of GAP-43-positive cells in the hippocampal CA1, CA3, and DG regions in the CUMS group was reduced (*p* < 0.01); the percent of GAP-43-positive cells in the hippocampal CA1, CA3, and DG regions in the CUMS group was lower than that of the FLU group and the H-HG group (*p* < 0.05). Additionally, HG upregulated NCAM and GAP-43 protein expression.

## 6. Discussion and Conclusions

Depression is a severe psychiatric illness with a high rate of clinical morbidity [31]. The side effects of antidepressants such as sexual dysfunction, weight gain, and other emotional disorders prompted medicinal chemists to search for safer and effective drugs without these side effects [32]. Recently, TCM herbs have received an increasing amount of attention [33]. Many common Chinese herbal formulas for treating depression have been reported over the years [13,14], but the effect of aqueous HG extract on depression has not been reported previously. Thus, we explored the effect of HG against depression using animal behaviors and the related mechanisms.

First, we detected the nuciferine in the HG aqueous extract using HPLC, and the result is consistent with our previous study [34]. We used the CUMS protocol to mimic the traits of depression, which were confirmed by the SPT, FST, TST, EPM, and OFT in mice in our behavioral studies. FLU is currently available on the market [35], and the present results are consistent with previous research [36]. The CUMS mouse model has been generally used to study the pathological mechanism of depressive disorder and screening antidepressant drugs [37]. Our study shows that HG can significantly alleviate depression-like symptoms in the CUMS mouse model.

The OFT and EPM tests are commonly used experimental methods to study the behavioral pharmacological effects of animals including fear, anxiety, and alertness [38]. By testing the OFT entries, distance, and duration in the center, the results showed that CUMS mice expressed significant depression-like behavior compared to the control mice (Figure 7). For EPM, we found that CUMS group mice spent obviously less time in the open arms, entered significantly fewer open arms, and went a shorter distance into the open arms compared to the CON group (Figure 6).

The FST and TST are two of the most commonly used depression models for evaluating antidepression activity in animals [39]. The enhanced immobility time in the FST and TST is suggestive of despair behavior. The CUMS model animals demonstrated a significant increase in immobility time in the FST/TST compared to the CON group (Figures 3(a)–3(c)). The SPT represents the change in depression-like behavior [40]. In our study, CUMS group mice showed a decreased preference for the sucrose solution compared to the CON group mice (Figure 3(d)). The results of the behavioral tests showed that CUMS can cause depression-like behavior.

The main findings of the RAM and MWM tests are that chronic restraint stress impairs spatial learning and memory and induces stress [41]. The RAM test can reflect the spatial memory ability of mice according to the analysis of the number of working memory errors and residence time in the correct feeding arms. The MWM test is a type of spatial memory and learning ability method according to the analysis of times crossing platform and mean escape latency. We revealed significant changes in the H-HG mice compared with the CUMS mice after HG treatment. In our test, memory and learning ability were evaluated by the RAM and the MWM test (Figures 4 and 5), and the results confirmed the antidepression function of HG.

NCAM is a crucial regulator of axon growth, fasciculation, and cell adhesion [42]. GAP-43 has also been shown to affect synaptic plasticity because it has been suggested to regulate presynaptic properties and to significantly enhance long-term potentiation (LTP) and learning [43]. It had been reported that phosphorylation of GAP-43 is substantial in NCAM-mediated neurite outgrowth via activation of the fibroblast growth factor (FGF) receptor [44]. NACM and GAP-43 expression is important for memory and learning ability. Synaptic plasticity is an important neural basis for learning and memory ability [45]. In our study, NCAM and GAP-43 expression in H-HG remarkably increased compared with the CUMS mice. This is consistent with our results. The study showed that HG may have an antidepressant effect by affecting neural structure plasticity.

**Figure 11:**
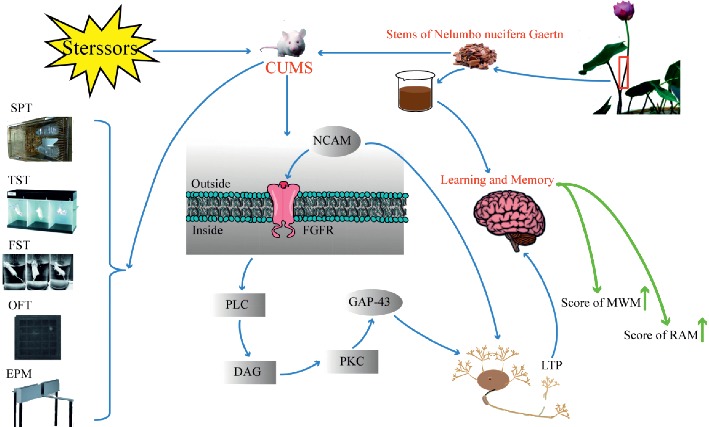
A schematic diagram of the rationale for the experimental design and the research results.

This research has provided a possible mechanism for the antidepressive action of HG and the improvement of learning and memory ability by observing hippocampal CA1, CA3, and DG regions as well as immunofluorescence and behavioral test results (Figure 11). However, further researches are needed to explore the effect of HG on the differences (or changes) of different hippocampal areas, and Western blot can also be used to precisely identify the target of the drug action.

In conclusion, our results suggest that HG can improve depression-like behavior in CUMS mice, which may be related to regulating the NCAM and GAP-43 expression levels in hippocampal neurons. Further tests are needed to show the antidepressant-like effect of HG and investigate the underlying mechanisms. Thus, these results confirm the notion that HG is beneficial for the treatment of depression; its exact molecular mechanism and clinical application require further investigation.

## Data Availability

The data used to support the findings of this study are available from the corresponding author upon request.
